# Social Determinants of Health, Diet, and Health Outcome

**DOI:** 10.3390/nu16213642

**Published:** 2024-10-26

**Authors:** Li Jiao

**Affiliations:** Baylor College of Medicine, Houston, TX 77030, USA; jiao@bcm.edu

## 1. Introduction

This Special Issue delves into the intricate relationship between social determinants of health (SDOHs), diet, and health outcomes through 13 articles authored by scholars from 10 countries across five continents. The research examines how various socioeconomic factors, such as poverty, education level, occupation, geographic location, race/ethnicity, place of birth, immigration status, refugee status, and disability, affect food insecurity, diet quality, and food choices among different populations, including school-aged children, pregnant women, and the elderly. In low- and middle-income countries, food insecurity is associated with poverty, job instability, refugee status, and disability. In the U.S., geographic location and occupation influence food insecurity and dietary choices. Five articles in this Special Issue address health outcomes, linking food insecurity and poor diet to issues such as malnutrition, anemia in children, obesity, metabolic disorders, and cardiovascular diseases. Other factors like culture, climate, reginal conflict, dental hygiene, and social networks also impact food availability and diet quality. Stress and nutritional illiteracy might mediate the relationship between SDOHs and health outcomes. The authors advocate for interventions through policy, regulation, and health education, particularly for pregnant women, children, and the elderly, to mitigate the negative impacts of SDOHs on health. Future research could investigate the broader impacts of SDOHs and diet on preventable chronic diseases. Overall, this Special Issue highlights the critical connections between SDOHs, food insecurity, and diet qualities, emphasizing the need for comprehensive intervention to reduce health disparities and promote population health.

## 2. Social Determinants of Health, Food Insecurity, and Health Outcomes

Social determinants of health (SDOHs) are the non-medical conditions in which people are born, grow, work, live, worship, and age. Food insecurity occurs when households are, at times, unable to acquire adequate food for one or more members due to insufficient resources, such as finance [1]. Numerous studies have examined the relationship between food insecurity and socioeconomic status (SES), identifying contributing factors, including poverty [2], unemployment [3], immigration status [4], refugee status [5], disability [6], limited access to grocery stores, lack of transportation [7], and lower education levels [8,9]. This Special Issue highlights the association between these factors and food insecurity in Maputo City, southern Mozambique. In Peru, women’s autonomy, immigration status, refugee status, and disability among Venezuelans were associated with unmet food needs. In the United States, children in urban areas were found to have a higher prevalence of food insecurity compared to their rural counterparts.

Malnutrition is a common consequence of food insecurity, particularly among the elderly, who may face challenges such as limited mobility and transportation. Malnutrition can lead to various health problems, including heart disease [10], type 2 diabetes, and certain cancers [11,12]. In Peru, low women autonomy was associated with a high prevalence of anemia in young children, highlighting this as an important social and public health issue in low- and middle-income countries (LMICs).

In summary, the findings from four studies in this Special Issue underscore the need for social inclusion, economic support, humanitarian aid, and improved access to safety nets for vulnerable populations in LMICs. Ensuring access to nutritious food is vital in reducing the burden of chronic diseases [13].

## 3. SDOHs, Dietary Quality, and Health Outcomes

**Figure 1:**
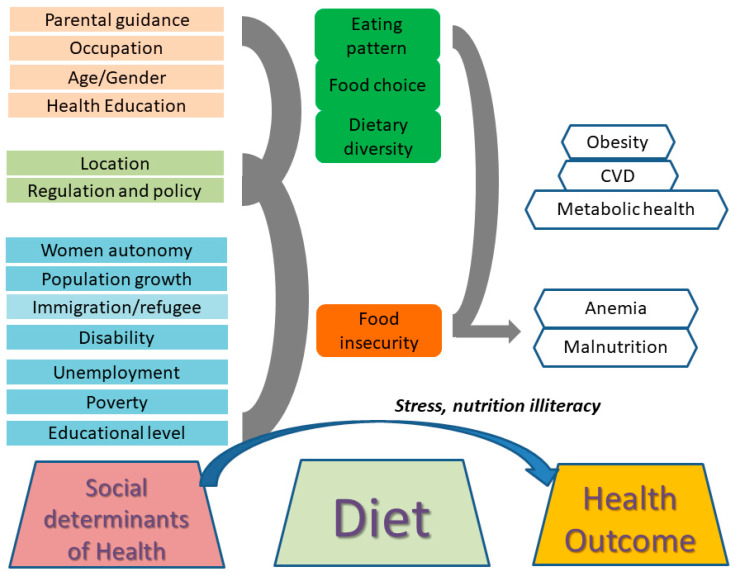
The SDOHs, diet, and health outcomes discussed in this special issue.

Several studies have investigated the relationship between SDOHs and dietary quality, diversity, and food choices, focusing on factors such as race/ethnicity, place of birth, occupation, education, and geographic location. Below is a summary of key findings (Figure 1).

Race/Ethnicity and place of birth. One study found that U.S.-born non-Hispanic African American adults had lower diet quality than their foreign-born counterparts. Furthermore, foreign-born African American who had lived in the U.S. for less than 10 years had better diets than those who had been in the country for a longer period of time. This study highlighted the need for further research on dietary acculturation among immigrants and health in the U.S. [14].

Occupation. Mansouri et al. found that the unpredictable nature of emergency medical services (EMSs) poses barriers to maintaining a healthy diet. The barriers included fatigue, heavy workloads, lack of meal breaks, reliance on convenience food, and limited food options at work [15]. A previous study showed that hospital workers who skipped meals tended to make less healthy dietary choices, with those skipping breakfast having lower diet quality. Understanding how different occupations influence dietary habits could offer new opportunities for promoting healthy choices at work.

Geographic location and education levels. A study in Indonesia revealed that rural districts had a higher prevalence of inadequate fruit and vegetable consumption compared to urban areas, especially among females and older adults. Additionally, residents of districts with lower education levels consumed less fruits and vegetables. However, in the U.S., no significant differences in diet quality were observed between urban and rural areas.

Health outcome. Several studies have linked dietary habits to health outcomes. For example, obesity has been associated with unhealthy food choices related to occupation, nutrition illiteracy, and skipping breakfast in Greek children. Those who skipped breakfast also had poorer cardiorespiratory fitness, weaker handgrip strength, and worse metabolic health [16].

## 4. Other Factors and Diet Quality

Other factors, including climate change, political instability, regional conflicts, and the COVID-19 pandemic, have exacerbated food insecurity in Maputo City, southern Mozambique. Additionally, skipping breakfast, poor dental health in the elderly, cultural practices like eating together or alone in Japan, and peer influence among EMS providers in the U.S. have also been shown to affect food insecurity and diet quality. In a previous study in Spain, skipping breakfast has been associated with lower SES [17], highlighting its potential as an SDOH indicator.

## 5. SDOHs, Stress, and Health Outcomes

This Special Issue suggests that SDOHs influence health outcomes through stress and dietary choices. For instance, EMS is associated with stress, fatigue, and irregular eating patterns [15], which, when combined with reliance on snack-based foods, can further increase the risk of obesity, metabolic disturbance, diabetes, and cardiovascular disease (CVD). A review article discussed that pregnancy as a stress status [18] is often associated with increased consumption of high-fat and high-sugar foods and lower intake of healthy foods [19]. Moreover, “stress eating” can increase the risk of CVD in later life.

In summary, stress, whether directly or through its influence on dietary choice, can elevate the risk of chronic diseases. Additionally, other SDOHs, such as job instability, can also act as a chronic stressor that adversely impact health [20].

## 6. Policy and Regulation

Several studies in this Special Issue emphasize the role of policy and regulation in promoting healthy diets. For example, regulatory measures in Chile significantly reduced the purchase of sugar-sweetened beverages and unhealthy foods, shifting consumer behavior toward healthier products. One study underscores the need for further understanding how policies, such as taxes on unhealthy foods and beverages, could reduce the consumption of unhealthy products. In the U.S., adjusting shift schedules and revising organizational policies could support healthier eating habits among EMS providers and potentially reduce employee turnover costs for agencies. In Indonesia, policy recommendations include improving access to diverse and affordable fruit and vegetable options in rural and low-income areas and launching public health campaigns targeting females, older adults, and those with lower education levels. A Japanese study found that among older adults, personal dental health and cultural factors play a significant role in shaping dietary habits. This finding suggests that policymakers and regulators should consider cultural and health factors when developing nutritional guidelines for diverse elderly populations.

## 7. Potential Intervention: When and Where

Interventions targeting SDOHs aim to reduce the adverse effects of food and nutritional insecurity on health outcomes. Chronic non-communicable diseases (NCDs) often develop over time, making early education on nutrition literacy crucial. Several studies highlight the importance of influencing children through both parents and schools. For example, a Polish study found that childhood food restrictions were linked to lower levels of intuitive eating and higher levels of restrained eating in adulthood. Another study emphasized the critical role schools play in shaping children’s health habits when healthy eating is not nurtured at home, as seen in obesity research in Saudi Arabia. This approach not only helps prevent childhood obesity but also has long-term benefits for the health of the population.

A review article in this Special Issue advocates for interventions during pregnancy to help women manage stress and adopt healthy dietary habit and lifestyles, which can prevent cardiovascular diseases later in life. Nutritional intervention during pregnancy supports fetal growth and contributes to epigenetic programming in the fetus [21], potentially influencing the risk of NCD in adults. Maternal nutrition’s impact on offspring’s risk of NCD in adulthood has been examined [22]. Furthermore, maternal diet can have a transgenerational effect and may profoundly impact the health of the population [23]. On the other hand, a mother’s knowledge about nutrition can also be passed down to their children. Recently, in 2024, the U.S. Department of Agriculture made permanent increases to the cash value benefit for fruits and vegetables in the Special Supplemental Nutrition Program for Women, Infants, and Children (WIC) program. Such policies are crucial for improving health outcomes, closing nutrition gaps, and impacting health beyond the current generation.

In summary, promoting nutrition literacy and healthy eating habits should begin as early as possible as these interventions can significantly improve the health of the population.

## 8. Conclusions

SDOHs are recognized for having multiple factors related to health outcomes, such as the environment and access to health care. Diet is one pathway through which socioeconomic factors can impact health. Further research is needed to explore how social, political, and cultural factors relate to diet and health. Identifying effective intervention targets can help address health disparities due to SDOHs. Governmental and public agencies should promote collaboration across sectors—such as health, economic support, humanitarian aid, education, and municipalities—alongside families, schools, and communities to enhance healthy eating and reduce food insecurity. The collected studies have also shown gender differences in SDOHs and dietary habits and emphasize the need for a holistic and culture-sensitive approach to nutrition policy tailored to specific populations.
